# Blood-Based Gene Expression Profiles Models for Classification of Subsyndromal Symptomatic Depression and Major Depressive Disorder

**DOI:** 10.1371/journal.pone.0031283

**Published:** 2012-02-13

**Authors:** Zhenghui Yi, Zezhi Li, Shunying Yu, Chengmei Yuan, Wu Hong, Zuowei Wang, Jian Cui, Tieliu Shi, Yiru Fang

**Affiliations:** 1 Division of Mood Disorders, Shanghai Mental Health Center, Shanghai Jiao Tong University School of Medicine, Shanghai, China; 2 Department of Genetics, Shanghai Mental Health Center, Shanghai Jiao Tong University School of Medicine, Shanghai, China; 3 The Center for Bioinformatics and Institute of Biomedical Sciences, The College of Life Sciences, East China Normal University, Shanghai, China; 4 Shanghai Information Center for Life Sciences, Chinese Academy of Sciences, Shanghai, China; Baylor College of Medicine, United States of America

## Abstract

Subsyndromal symptomatic depression (SSD) is a subtype of subthreshold depressive and also lead to significant psychosocial functional impairment as same as major depressive disorder (MDD). Several studies have suggested that SSD is a transitory phenomena in the depression spectrum and is thus considered a subtype of depression. However, the pathophysioloy of depression remain largely obscure and studies on SSD are limited. The present study compared the expression profile and made the classification with the leukocytes by using whole-genome cRNA microarrays among drug-free first-episode subjects with SSD, MDD, and matched controls (8 subjects in each group). Support vector machines (SVMs) were utilized for training and testing on candidate signature expression profiles from signature selection step. Firstly, we identified 63 differentially expressed SSD signatures in contrast to control (P< = 5.0E-4) and 30 differentially expressed MDD signatures in contrast to control, respectively. Then, 123 gene signatures were identified with significantly differential expression level between SSD and MDD. Secondly, in order to conduct priority selection for biomarkers for SSD and MDD together, we selected top gene signatures from each group of pair-wise comparison results, and merged the signatures together to generate better profiles used for clearly classify SSD and MDD sets in the same time. In details, we tried different combination of signatures from the three pair-wise compartmental results and finally determined 48 gene expression signatures with 100% accuracy. Our finding suggested that SSD and MDD did not exhibit the same expressed genome signature with peripheral blood leukocyte, and blood cell–derived RNA of these 48 gene models may have significant value for performing diagnostic functions and classifying SSD, MDD, and healthy controls.

## Introduction

Depression affects about 10% of the population at some point in their life and is the leading cause of disability across the world [1]. Lacking specific objective findings, depression is often missed or undiagnosed [2] and studies have focused on subthreshold depressive [3]–[5]. At present, some types of subthreshold depressive, including dysthymia, minor depression (MinD) and recurrent brief depression (RBD), are described in the Diagnostic and Statistical Manual of Mental Disorders, 4th edition (DSM-IV) [6]. However, approximately two-thirds to three-fourths of all subthreshold depressive patients with psychosocial functional impairment did not meet any criteria of DSM-IV [7]. Consequently, the concept of subsyndromal symptomatic depression (SSD) was introduced by Judd in 1994, which is characterized by two or more depressive symptoms, but without depressed mood or anhedonia, lasting for at least 2 weeks accompanied with social dysfunction, and does not meet the criteria for MDD, dysthymia, MinD or RBD [7]–[8]. Convergent evidence has identified that SSD is a common depressive status that affects different ethnic populations [7], [9]–[11] and to which we must pay more attention. However, litter research has been conducted on the biological basis of SSD.

Although the pathophysioloy of depression spectrum remain largely obscure, it has been reported that patients with SSD and MDD have similar family history, and their first-degree relatives have a high risk of comorbidity of depression and alcohol dependence, which implies that these two disorders could share genetic bases^12^. Furthermore, several follow-up studies have suggested that SSD is a transitory phenomena in the depression spectrum and is thus considered a subtype of depression [10], [13]–[14]. In addition, previous twin data supported that unipolar depression had a modest heritability [15]. SSD and MDD, which have different depressive symptoms, may be different subtypes of depression and have different phenotype at gene expression levels.

With the sequence of the human genome being publicly available since February 2001, an array of novel research tools, such as gene expression microarray, have become available that may yield unbiased, hypothesis-free insight into the pathophysiologic underpinnings of this disorder [16]. The application of high-throughput gene expression profiling to MDD in humans has mostly been restricted to postmortem brain tissue, typically sampled many decades after the critical time frame during which the initial molecular processes underlying the onset and development of disease have occurred, with methodological challenges including decades of cumulative drug exposure and postmortem artifacts [17]–[21]. Convincing evidences indicated that depression affects the entire organ systems, including endocrinological, immunological and autonomic nervous systems, through the interaction between the brain and the body [22]. Circulating blood comprises a highly complex system that communicates with every tissue and organ in the body. Peripheral blood cells share more than 80% of the transcriptome with nine tissues: brain, colon, heart, kidney, liver, lung, prostate, spleen, and stomach, and the expression levels of many classes of biological processes have been shown to be comparable between whole blood and prefrontal cortex [23]–[24]. Indeed there is considerable communication between the immune system and the central nervous system (CNS). Many cytokine receptors have been located within the CNS, and interleukin-2 mRNA and T-cell receptors have been specifically detected in neurons [25]. Lymphocytes also express several neurotransmitter and hormone receptors, including dopamine, cholinergic, and serotonergic receptors and glucocorticoid and mineralocorticoid receptors and their chaperones [26]. Lymphocytes are directly influenced by glucocorticoids and catecholamines, and these two systems are perturbed in MDD [27]. The circulating blood may act as a “sentinel tissue” that can reflect states of health or disease within the body. Some studies successfully discriminated between control subjects and physical disease patients via detection of the expression of “tissue-specific” genes in circulating blood [28]–[30]. Blood based gene expression diagnostics could be applied to the study of psychiatric disorders for which human brain tissue biopsy samples are unavailable.

Disease development is a systematic and dynamic processes influenced by environment factors and genetic factors, together. Computational and systems biology have greatly facilitate the disease studies from transcriptomes by using microarray technology [31]. Based on gene expression profiles, thousands of genes can be featured simultaneously in different conditions or clinical phenotypes [32]. Scientists have utilized high-throughput technology and computational approach to built disease models and classify disease state.

With gene features made on microarray accumulated by technology developing, many psychiatric disorder studies are also applied by the high-throughput technology with bioinformatics analysis. Tsuang et.,al have assessed the validity of blood-based gene expression profiles for the classification of schizophrenia and bipolar disorder [33]. Segman et.,al found gene expression signatures that could differentiate between women prone to postpartum depression [34]. Le-Niculuscu et.,al demonstrated that peripheral blood gene expression profiles could offer an unexpectedly informative insight into brain function and disease state [35]. Most recently, Spijker et.,al also found that gene expression profiles could be used as a blood marker of MDD, and careful independent validation has been carried out to prove their results [36].

Thus, in order to develop the potential peripheral blood lymphocytes gene expression signature models which can classify MDD, SSD, and healthy controls, whole-genome cRNA microarray analysis of lymphocytes were performed in this study.

## Results

### Pathway analysis and GO analysis results for SSD gene expression signatures

**Figure 1:**
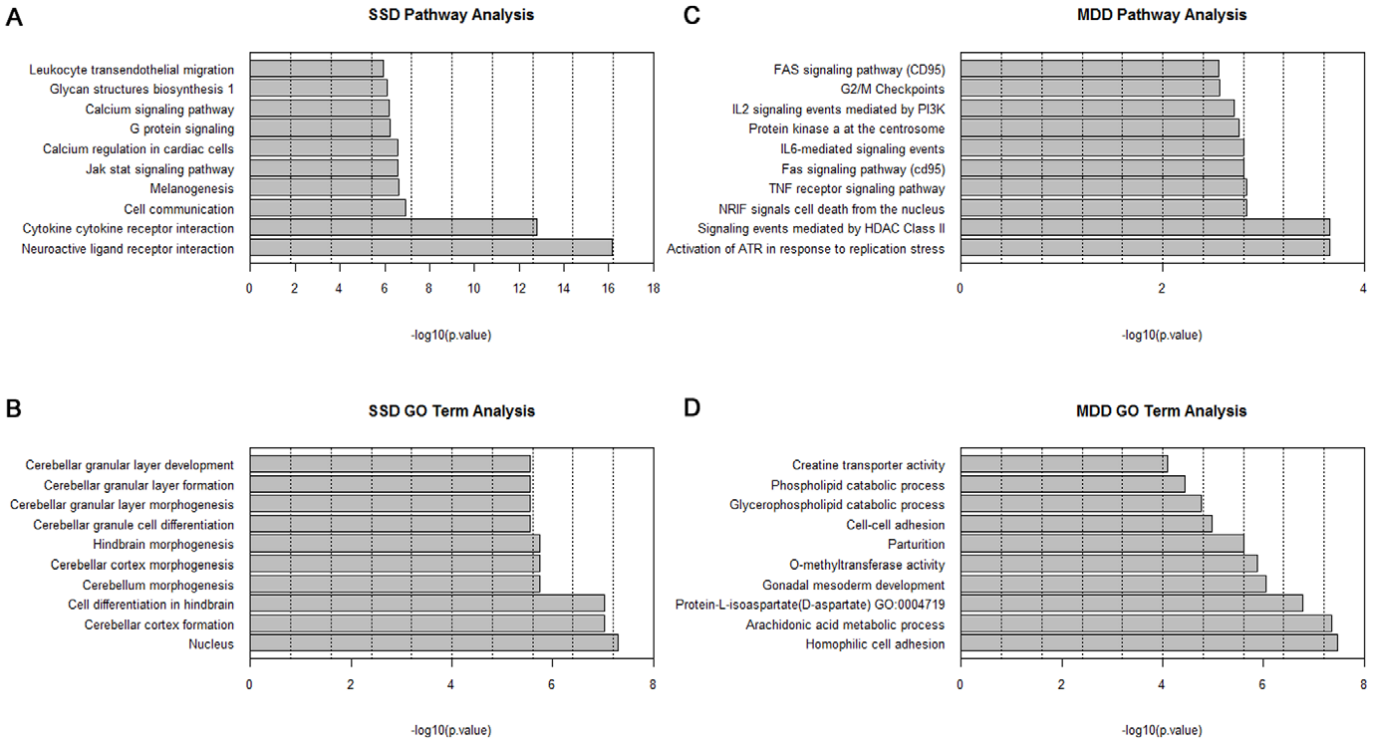
Functional annotation of the DEGs in SSD and MDD. (A) and (C) Pathway analysis of SSD-associated and MDD-associated genes respectively. The y-axis shows the KEGG Pathway terms, and the x-axis shows the enrichment significance P-values for the top 10 enriched Pathway terms. (B) and (D) GO analysis of SSD-associated and MDD-associated genes respectively. The y-axis shows the GO terms, and the x-axis shows the enrichment significance P-values for the top 10 enriched GO terms. Term GO:0004719 remarks the function of protein-L-isoaspartate (D-aspartate) O-methyltransferase activity. MDD: Major depression disorder; SSD: Subsyndromal symptomatic depression.

For SSD gene expression signatures, we detected 1,456 differential expressed genes between SSD and healthy controls, in which 753 genes are up regulated and 703 genes are down regulated (adjusted p<0.01), which enriched in 47 pathways (P<0.01). Most of genes involved in several functional related to signaling pathways, including neuroactive ligand receptor interaction, JAK and STAT signaling pathway, G protein signaling, calcium signaling pathway, insulin signaling pathway, GNRH signaling pathway, Wnt signaling pathway and MAPK signaling pathway etc. Cellular communication and cell structure organization were also important in SSD process, such as apoptosis, cell adhesion molecules, tight junction, focal adhesion. The DEG also act in several biosynthesis and metabolism pathways, like oxidative phsphorylation, metabolism of xenobiotic by cytochrome P450, purine metabolism, glycerlipid metabolism, glycan structures biosynthesis, glycerolipid metabolism, starch and sucrose metabolism. We also found that SSD signatures participate in immunity process, antigen processing, leukocyte transendothelial migration, natural killer cell mediated cytototoxicity and cytokine-cytokine receptor interaction (Figure 1A). GO analysis indicate that SSD gene signatures correlate with cerebellar cortex morphogenesis, cerebellar granular layer development, hydrolase activity, GTPase and ATPase activity, S phase and M phase of mitotic cell cycle and tissue regeneration, etc. (Figure 1B).

### Pathway analysis and GO analysis results for MDD gene expression signatures

Based on pre-processed microarray profile, we identified 149 differential genes between MDD patients and controls with 95 upregulated and 54 down-regulated (adjusted P<0.01), 20 of which were identified between SSD and control. These differential genes enriched in 53 pathways, 2 of which also were identified in SSD. Signaling pathways active in MDD include activation of ATR in response to replication stress, NRIF signals cell death from the nucleus, fas signaling pathway, p53-Independent G1/S DNA damage checkpoint and Nicotinamide salvaging, and EGF signaling pathway etc. We noticed that many MDD signatures involves several immunity process, such as T cell receptor signaling pathway and JNK signaling in the CD4+ TCR pathway, IL2-mediated signaling events, IL1 signaling and IL6-mediated signaling events and Calcium signaling in the CD4+ TCR pathway and TCR signaling in CD4+ T cells. In MDD subjects, more biosynthesis and metabolism pathway are identified, involveing Vitamin B5 (pantothenate) metabolism, coenzyme A biosynthesis and metabolism of water-soluble vitamins and cofactors. Comparing with former results of SSD signatures, we found that several pathways were shared in MDD and SSD process, including cell cycle controls and Cell Cycle Checkpoints, like G2/M Checkpoints and Wnt signaling. We noticed that MDD-specific functions or pathways compared with SSD were activation of ATR pathway in response to replication stress, NRIF signals cell death from the nucleus, fas signaling pathway, immunity pathway about IL2 signaling events mediated by PI3K and IL1 singaling events. Meanwhile, SSD-specific pathways contain cytokine-cytokine receptor interaction, GPCRDB class A rhodopsin like, MAPK signaling pathway, neuroactive ligand receptor interaction, calcium signaling pathway, breast cancer estrogen signaling pathway, purine metabolism, insulin signaling pathway, cell adhesion molecules and Toll like receptor signaling pathway(Figure 1C). Alternatively, GO analysis for MDD signatures, 29 of which also were identified in SSD, convinced us that most significant functions (P<0.01) are active in immunity reactions involving pro-B cell differentiation, negative regulation of antigen processing, positive regulation of leukocyte migration and plasminogen activation. Other functions of these genes involve engulfment of apoptotic cell, fibrinogen binding, lymphoid progenitor cell differentiation and immunoglobulin V(D)J recombination and mitotic cell cycle controls (S phase), DNA ligation involved in DNA repair and somatic cell DNA recombination, etc. al. (Figure 1D).

### Gene expression profiles for classification of subsyndromal symptomatic depression and major depressive disorder

**Figure 2:**
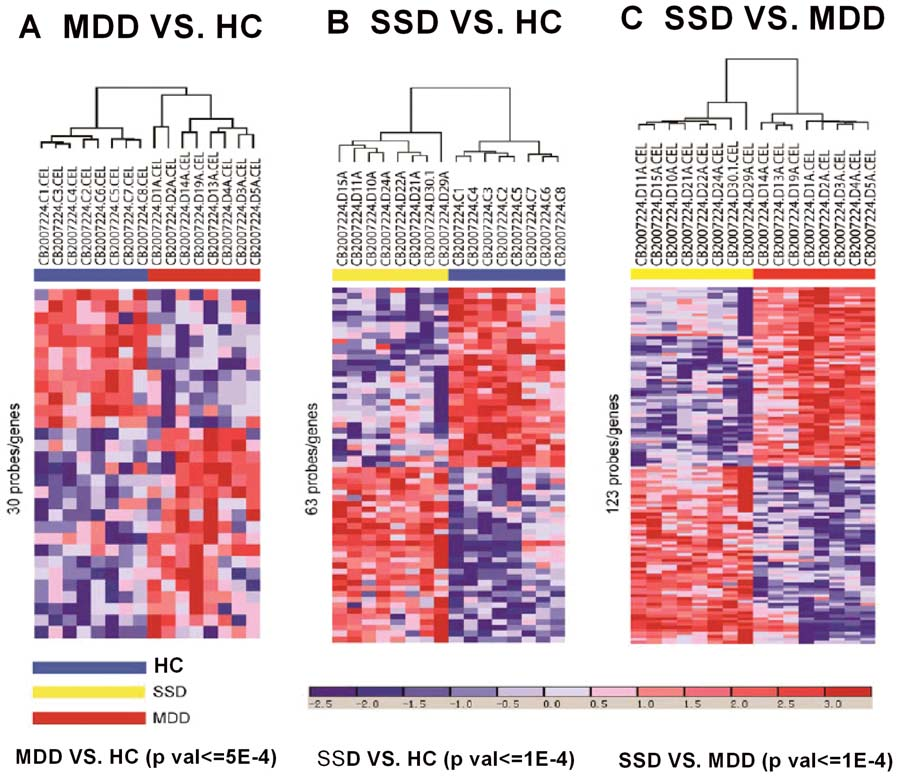
Biomarkers differentiation efficiency among MDD group, SDD group and HC. (A) Complete linkage clustering analysis with 16 samples using 30 biomarkers under the criteria of adjusted.P< = 5E-4 between MDD and HC. (B) Complete linkage clustering analysis with 16 samples using 63 biomarkers under the criteria of adjusted.P< = 1E-4 between SSD and HC. (C) Complete linkage clustering analysis with 16 samples using 123 biomarkers under the criteria of adjusted.P< = 1E-4 between SSD and MDD. MDD: Major depression disorder; SSD: Subsyndromal symptomatic depression; HC: Healthy controls.

In order to filter out most of false positives and select most potential biomarkers, we applied strict threshold on the same pair-wise comparisons among SSD, MDD and controls, and identified 63 differentially expressed SSD signatures in contrast to controls (adjusted P< = 1.0E-4) and 30 differentially expressed MDD signatures in contrast to controls (adjusted P< = 5.0E-4), respectively. Then, 123 gene signatures were identified with significantly differential expression level between SSD and MDD (adjusted P< = 1.0E-4). Unsupervised hierarchal clustering analysis by using Euclidean distance and complete linkage clustering method was conducted on three more potential groups of DEGs. The results showed clearly that genes differentially expressed in the peripheral blood lymphocytes were capable of differentiating MDD group, SSD group and healthy controls, separately (Fig. 2 A, B, C).

To evaluate the predictive performances of SSD and MDD signatures respectively, we utilized SVM with Linear Kernel to build disorder models. For 63 SSD signatures (adjusted P< = 1.0E-4), total instances (8 SSD and 8 control instances, respectively) were correctly classified. Similarly, for 30 MDD signatures (adjusted P< = 5.0E-4), and 123 DEGs signatures (adjusted P< = 1.0E-4) can also clearly classified 8 MDD and 8 SSD instances.

For SSD gene signatures, we detected 1,456 differential expressed genes between SSD and control; For MDD gene signatures, we identified 149 differential genes between MDD patients and controls d. Among these genes, there are only 20 different genes between SSD and MDD.

Furthermore, in order to conduct priority selection for biomarkers for SSD and MDD together, we selected top gene signatures from each group of pair-wise comparison results, and merged the signatures together to generate better profiles used for clearly classify SSD and MDD states in the same time. In details, we tried different combination of signatures from top ranked signatures in the three pair-wise compartmental results and finally determined 48 gene expression signatures (Table 1). To maintain the robustness of SSD-MDD disorder model, the predictive power was evaluated using cross validation, which randomly took 9/10 samples used for training and remaining 1/10 as internal testing validation.

**Table 1:** Mostly potential signatures of gene expression profiles models for classification of MDD and SSD.

Probe	Gene Symbol	SSD vs. HC	MDD vs. HC	MDD vs. SSD
		FC (adjusted p<0.0001)	FC (adjusted p<0.0005)	FC (adjusted p<0.0001)
202123_s_at	ABL1	0.741304049	1.071540957	1.445481053
203142_s_at	AP3B1	0.785262839	1.163049415	1.481095700
212645_x_at	BRE	0.622357591	0.989984919	1.590701125
213986_s_at	C19orf6	0.479842399	1.004084118	2.092528964
200723_s_at	CAPRIN1	0.716725182	1.016565288	1.418347389
200952_s_at	CCND2	0.432720956	0.982303164	2.270061461
211188_at	CD84	0.521512199	0.928367596	1.780145501
1554339_a_at	COG3	0.357536796	0.991125007	2.772092322
204925_at	CTNS	0.650193383	0.962500784	1.480330023
201275_at	FDPS	1.312775229	0.929569711	0.708095103
1555407_s_at	FGD3	0.459952028	1.051968262	2.287126038
204867_at	GCHFR	1.417582619	1.159141932	0.817689154
1554356_at	GINS4	0.943637323	0.557990569	0.591318885
204553_x_at	INPP4A	0.727800115	0.966193858	1.327553869
206078_at	KALRN	2.421891723	0.831693595	0.343406597
225642_at	KTI12	1.032925752	1.217211291	1.178411218
1565406_a_at	LHX9	3.616566285	4.484406509	1.239962483
212535_at	MEF2A	0.739248358	0.967245398	1.308417379
1557172_x_at	NEK8	1.496780327	0.827608072	0.552925541
200875_s_at	NOP56	1.361209547	0.941029044	0.691318281
202647_s_at	NRAS	0.706715422	1.122941274	1.588958217
216422_at	PA2G4	2.674676802	2.203654734	0.823895707
1557777_at	PDE6B	0.318473762	0.978799188	3.073406053
1554508_at	PIK3AP1	0.651835063	1.287048315	1.974499972
1567214_a_at	PNN	1.276179391	0.992966418	0.778077462
204842_x_at	PRKAR2A	1.453510563	0.905753493	0.623148891
209685_s_at	PRKCB	1.459071747	1.094764429	0.750315693
204021_s_at	PURA	2.021380345	1.105199279	0.546754737
212120_at	RHOQ	0.618194385	0.928620496	1.502149676
200089_s_at	RPL4	1.238633645	0.961953034	0.776624337
226923_at	SCFD2	0.794178375	0.719803168	0.906349494
1552812_a_at	SENP1	0.675377839	1.137970532	1.684939105
204019_s_at	SH3YL1	0.757933305	0.658471379	0.868772193
202855_s_at	SLC16A3	0.504525282	1.003367055	1.988734935
1552792_at	SOCS4	0.424980413	0.877342521	2.064430488
205026_at	STAT5B	0.497586762	0.987611266	1.984802134
205520_at	STRN	0.476780581	1.116717124	2.342203456
203611_at	TERF2	0.799885457	1.051562728	1.314641639
200804_at	TMBIM6	1.362494303	1.173043411	0.860952892
212282_at	TMEM97	0.633075615	0.722453817	1.141180927
201796_s_at	VARS	0.539085621	1.680791788	3.117856841
1552737_s_at	WWP2	0.324152671	0.963600812	2.972675826
225072_at	ZCCHC3	1.512482696	3.071112709	2.030510971
1554769_at	ZNF785	1.899359591	0.781703552	0.411561641
1553704_x_at	ZNF791	1.393734556	0.892755221	0.640548961
202848_s_at	GRK6	0.543637323	0.357990569	0.658509918
206382_s_at	BDNF	0.407145759	0.358749192	0.881132014
202343_x_at	COX5B	0.557536796	0.391125007	0.701523217

MDD: Major depression disorder; SSD: Subsyndromal symptomatic depression; HC: Healthy controls.

When 54 probesets (48 genes) were chosen as biomarkers, we obtained the best predictive performances with 100% accuracy and 100% TPR (leave-one-off validation). Leave-one-off validation refers to that we used n-1 sample to train model and used another sample to test the model. Total 24 MDD, SSD and control samples were separated into train and test profiles in 24 times and train/test the data using leave-one-off method. Finally, we collected the predictions for each sample, and obtained 100% predictive performance (Table 2).

**Table 2:** Predictive performances of disorder model.

Biomarker Number	Class label	TPR	FPR	Accuracy	ROC area
30(10*3)	HC	1	0.063	0.889	0.969
	SSD	0.875	0.063	0.875	0.883
	MDD	0.875	0	1	0.969
	Weighted	0.917	0.042	0.921	0.94
45(15*3)	HC	1	0.063	0.889	0.969
	SSD	0.875	0	1	0.965
	MDD	1	0	1	1
	Weighted	0.958	0.021	0.963	0.978
54(18*3)	HC	1	0	1	1
	SSD	1	0	1	1
	MDD	1	0	1	1
	Weighted	0.958	0.021	0.963	0.978
60(20*3)	HC	1	0.063	0.889	0.969
	SSD	0.875	0	1	0.965
	MDD	1	0	1	1
	Weighted	0.958	0.021	0.963	0.978
75(25*3)	HC	1	0.063	0.889	0.969
	SSD	0.875	0	1	0.969
	MDD	1	0	1	1
	Weighted	0.958	0.021	0.963	0.979
90(30*3)	HC	1	0.063	0.889	0.969
	SSD	0.875	0	1	0.973
	MDD	1	0	1	1
	Weighted	0.958	0.021	0.963	0.98

MDD: Major depression disorder; SSD: Subsyndromal symptomatic depression; HC: Healthy controls.

Then, we interrogated the pathways and biological functions about these mostly potential biomarkers for MDD and SSD, together. Pathway analysis demonstrates that the potential 48 gene biomarkers involved in insulin signaling pathway, signaling by NGF, ErbB signaling pathway, neurotrophin signaling pathway, cell surface interactions at the vascular wall, NRAGE signals death through JNK, Rho GTPase cycle, and G alpha signaling pathway (P value<0.05) (Table 3). Also, GO analysis shows consistency with pathway analysis results. Besides, PURA and TERF2 both function about telomeric DNA binding and single strand DNA binding, and DNA replication. SLC16A3 and CTNS act in the directed movement of carboxylic acids into, out of, within or between cells. FGD3 and KALRN participate in Stimulates the exchange of guanyl nucleotides by a GTPase. Under normal cellular physiological conditions, the concentration of GTP is higher than that of GDP, favoring the replacement of GDP by GTP in association with the GTPase. Also, FGD3, KALRN and RHOQ involve in Rho GTPase cycle. Other signatures also correlated with Cell death signalling via NRAGE, NRIF and NADE, Jak-STAT signaling pathway, B cell receptor signaling pathway, and p75 NTR receptor-mediated signaling (Table 4).

**Table 3:** Mostly potential pathways of disorder model biomarker.

Gene Set Name	Genes in Gene Set (K)	Description	Genes in Overlap (k)	k/K	p value
KEGG insulin signaling pathway	137	Insulin signaling pathway	4	0.0292	1.15E-02
KEGG chronic myeloid leukemia	73	Chronic myeloid leukemia	3	0.0411	1.15E-02
Reactome signaling by NGF	215	Genes involved in Signalling by NGF	5	0.0233	1.20E-02
Reactome myogenessis	29	Genes involved in MyoGenessis	2	0.069	1.49E-02
KEGG ERBB signaling pathway	87	ErbB signaling pathway	3	0.0345	1.83E-02
Reactome down stream signal transduction	35	Genes involved in Down-stream signal transduction	2	0.0571	2.12E-02
Reactome cell surface interactions at the vascular wall	94	Genes involved in Cell surface interactions at the vascular wall	3	0.0319	2.25E-02
Reactome NRAGE signals death through JNK	47	Genes involved in NRAGE signals death through JNK	2	0.0426	3.68E-02
Reactome RHO GTPase_cycle	124	Genes involved in Rho GTPase cycle	3	0.0242	4.55E-02
Reactome G Alpha 12_13 signalling events	54	Genes involved in G alpha (12/13) signalling events	2	0.037	4.73E-02
KEGG neurotrophin signaling pathway	126	Neurotrophin signaling pathway	3	0.0238	4.74E-02
Biocarta PPARA pathway	58	Mechanism of Gene Regulation by Peroxisome Proliferators via PPARa(alpha)	2	0.0345	5.38E-02
KEGG acute myeloid leukemia	60	Acute myeloid leukemia	2	0.0333	5.71E-02
Reactome cell death signaling via NRAGE NRIF and NADE	61	Genes involved in Cell death signalling via NRAGE, NRIF and NADE	2	0.0328	5.88E-02
Reactome signaling by PDGF	64	Genes involved in Signaling by PDGF	2	0.0312	6.40E-02
KEGG JAK_STAT signaling pathway	155	Jak-STAT signaling pathway	3	0.0194	7.80E-02
KEGG B_cell receptor signaling pathway	75	B cell receptor signaling pathway	2	0.0267	8.43E-02
Reactome P75_NTR receptor mediated signalling	82	Genes involved in p75 NTR receptor-mediated signalling	2	0.0244	9.81E-02
KEGG chemokine signaling pathway	190	Chemokine signaling pathway	3	0.0158	1.24E-01
Reactome TRKA signaling from the plasma membrane	103	Genes involved in TRKA signalling from the plasma membrane	2	0.0194	1.43E-01

**Table 4:** Mostly potential GO functions of disorder model biomarker.

Gene Set Name	Genes in Gene Set (K)	Description	Genes in Overlap (k)	k/K	p value
Telomeric DNA binding	10	Genes annotated by the GO term GO:0042162. Interacting selectively with telomere-associated DNA, usually characterized by highly repetitive sequences.	2	0.2000	1.58E-03
Single stranded DNA binding	34	Genes annotated by the GO term GO:0003697. Interacting selectively with single-stranded DNA.	2	0.0588	1.80E-02
DNA replication	101	Genes annotated by the GO term GO:0006260. The process whereby new strands of DNA are synthesized. The template for replication can either be an existing DNA molecule or RNA.	3	0.0297	2.32E-02
Carboxylic acid transport	41	Genes annotated by the GO term GO:0046942. The directed movement of carboxylic acids into, out of, within or between cells. Carboxylic acids are organic acids containing one or more carboxyl (COOH) groups or anions (COO-).	2	0.0488	2.56E-02
Guanyl nucleotide exchange factor activity	42	Genes annotated by the GO term GO:0005085. Stimulates the exchange of guanyl nucleotides by a GTPase. Under normal cellular physiological conditions, the concentration of GTP is higher than that of GDP, favoring the replacement of GDP by GTP in association with the GTPase.	2	0.0476	2.67E-02
Organic acid transport	42	Genes annotated by the GO term GO:0015849. The directed movement of organic acids, any acidic compound containing carbon in covalent linkage, into, out of, within or between cells.	2	0.0476	2.67E-02
DNA_dependent DNA replication	55	Genes annotated by the GO term GO:0006261. The process whereby new strands of DNA are synthesized, using parental DNA as a template for the DNA-dependent DNA polymerases that synthesize the new strands.	2	0.0364	4.39E-02
Structure specific DNA binding	55	Genes annotated by the GO term GO:0043566. Interacting selectively with DNA of a specific structure or configuration e.g. triplex DNA binding or bent DNA binding.	2	0.0364	4.39E-02
Sequence specific DNA binding	57	Genes annotated by the GO term GO:0043565. Interacting selectively with DNA of a specific nucleotide composition, e.g. GC-rich DNA binding, or with a specific sequence motif or type of DNA e.g. promotor binding or rDNA binding.	2	0.0351	4.68E-02

### De novo cis-Regulatory element analysis results for candidate biomarkers of MDD and SSD

In order to investigate how the signatures for classifying three groups are regulated, we analyzed the cis-regulaotry elements co-occurring on the promoters of these genes. In details, STAT1 and STAT2 factor' binding motifs were detected on five MDD signatures' promoters (e.g. BDNF, MYB, THBS1, SORBS1, and SH3BGRL). In addition, we identified SRF binding motif on three gene promoters (e.g. THBS1, EGR1 and PODN). For SSD signatures, we identified transcriptional factor SREBP1 was correlated with eleven SSD signature genes (GNAS, MLL5, TOM1L1, DLGAP4, PTMA, NF1, ATP2B2, UNC13D, PDP2, CORO1A, and INPP4A). Most of these transcriptional factors are related with depression disorders as discussed below.

## Discussion

To our knowledge, this is the first study to compare the expression profile and make the classification with the leukocytes by using whole-genome cRNA microarrays among patients with SSD, major depressive disorder (MDD) and controls. We found that SSD and MDD had different blood-based gene expression signature, and the differential expressed genes of SSD were about 10 times of MDD, but there are only 20 overlapping differential expressed genes between SSD and MDD. Pathway analysis for SSD gene signatures showed that differential expressed genes enriched in 47 pathways, and most pathways were involved in regulation of DNA replication, IL2 signaling events mediated by STAT5, and Wnt signaling pathway, etc. For MDD gene signatures, the results of pathway analysis suggested that differential expressed genes enriched in 53 pathways, 2 of which also were identified in SSD, including MAPK signaling pathway and Wnt signaling pathway. Although the relationship between SSD and MDD is unclear, previous follow-up studies have showed that SSD was a subtype of depression and a transitory phenomenon in depression spectrum with a high likelihood of transition to MDD [10], [12]–[13]. It indicates that the genes involving in these two pathways maybe point to pathogenetically relevant underlying molecular processes of depression.

Genetic manipulation of the MAPK pathway, one of the neurotrophin signaling pathways, has received much attention, which postulated that the dysfunction of this pathway played a key role in the pathophysiology of mood disorders [37], especially in depression-like behavior [38]. Previous data also have shown that acute systemic blockade of MAPK signaling contributes to a depressive-like phenotype and blocks actions of antidepressants in animal models of depression [39].

Wnt signaling pathways have been implicated in various physiological functions, such as cell fate determination, cell and tissue polarity, synaptogenesis, dendritic morphogenesis, and axon remodeling. Moreover, abnormal Wnt signaling has been implicated in mood disorder. The relationship between Wnt signaling pathway genes and mood disorders has been reported in several genetic association studies. A study showed that alteration of hippocampal microRNA levels following chronic treatment with mood stabilizers is caused by effectors in the canonical Wnt signaling pathway. Gene expression-profiling of hippocampal subfields has also revealed altered expression of several genes related to Wnt signaling in bipolar disorder patients. Another study supports that the canonical Wnt signaling pathway and related substrates play a role in MDD. Wnt signaling pathway also has been considered relevant to the antidepressant effects, and Wnt2 expression and signaling is a common target of antidepressants and that increased Wnt2 is sufficient to produce antidepressant effects.

Moreover, patients with MDD have depressed mood or anhedonia but SSD have not, so differential expression of genes involving in other 51 pathways in MDD may be correlate with the underlying pathological mechanism of the symptom of depressed mood or anhedonia. Our unsupervised hierarchal clustering analysis showed obviously that each disease state exhibited a unique expressed genome signature except the genes involving in MAPK and Wnt pathways, which suggesting that these two diseases may be two different phenotypes in depression spectrum by respective gene signatures. Furthermore, genes differential expression among SSD group, MDD group, and healthy controls allowed us to discriminate among these three groups. It suggested that blood-derived RNA may potentially be used as a diagnostic tool for SSD and/or MDD, as long as the correct subsets of genes are employed. Blood profiling may also allow identification of differentially expressed genes that are involved in the pathophysiology of these disorders. To select the most potential biomarkers for differentiating these three groups, we combined top differential expressed genes from each set of gene expression signatures, then trained and tested the multiple combinatorial gene signatures from pair-wise comparison groups by using support vector machine classifier. Finally 48 gene expression signatures were determined. Samples can be grouped together according to the similarity of the expression levels of these 48 genes which suggested that different levels of gene expression may reflect different disease states. Among differential genes, BDNF, COX5B, GRK6 are the most significantly differential genes.

We comprehensively analyzed gene functions and pathway for the candidate biomarkers of SSD and MDD and found that potential biomarkers act in some pathways which have been found associated with function of CNS and implicated in depression, including insulin signaling pathway, signaling by NGF, ErbB signaling pathway and neurotrophin signaling pathway. We also found most of them were not reported the relationship with depression, such as cell surface interactions at the vascular wall, NRAGE signals death through JNK, Rho GTPase cycle, and G alpha signaling pathway, etc. al.

Some studies showed that there was a positive association between depressive disorder and insulin resistance due to dysregulation of insulin secretion or insulin receptor signaling. Otherwise, various functions for insulin receptor signaling in the brain have been suggested in normal neurophysiology, such as insulin receptor signaling maybe play a important role in synaptic plasticity and cognitive function,and several lines of work in both laboratory animals and humans suggest that when neurons in cognitive brain regions such as the hippocampus and cerebral cortex do not make enough insulin or cannot respond to insulin properly, everything from very mild memory loss to severe neorodegenerative diseases can result. Dysregulation of insulin secretion or insulin receptor signaling has also been reported in serious mental illnesses, such as Alzheimer's disease. Patients with depression also have some cognitive function problems and maybe have differential expression of genes involving in insulin signaling pathway.

It has been suggested that neuronal atrophy or destruction in the hippocampus and cortex is involved in the pathogenesis of depression. The neurotrophin systems modulate neuronal plasticity, inhibit cell death cascades and increase cell survival proteins that are responsible for proliferation and maintenance of central nervous system neurons. Thus the dysregulation of the neurotrophin systems, such as differential expression of genes involving in signaling by NGF (nerve growth factorA) and neurotrophin signaling pathway, may be involved in the pathophysiology of depression.

Transgenic mouse experiments have confirmed that the block of erbB signaling pathway will result in the change of OL number and morphology, reducing the thickness of myelin and the transmission rate of CNS axons [40]. The abnormal expression of ERBB (epidermal growth factor receptor, EGFR, epidermal growth factor receptor) signaling pathway can lead to oligodendrocytes (OL) abnormalities, which results in dopaminergic dysfunction, and it may be associated with depression [41]–[42].

The results of analysis of the cis-regulaotry elements co-occurring on the promoters of these genes showed that STAT1 and STAT2 factors were detected on five MDD signatures' promoters (e.g. BDNF, MYB, THBS1, SORBS1, and SH3BGRL). Especially, STAT1 mediates the autoimmune and inflammatory functions, and STAT2 mediates the virus protection function. From previous investigation about the immune cell specificity of activation programs induced by a major component of cell-mediated immunity, the transcriptional activators STAT1 were significantly induced in CD4+ and CD8+ T cells, B cells and monocytes [43]. Depression phenotypes are also correlated with immunity reactions reflected from blood transcriptomes [44]. In addition, we identified SRF binding motif on three gene promoters (e.g. THBS1, EGR1 and PODN). Up to now, there was no study about the relationship between SRF binding motif and depression. For SSD signatures, we identified transcriptional factor SREBF1 was correlated with eleven SSD signature genes (GNAS, MLL5, TOM1L1, DLGAP4, PTMA, NF1, ATP2B2, UNC13D, PDP2, CORO1A, and INPP4A). Several studies have reported the importance of SREBF1 and SREBF2 factors in the lipid biosynthesis and their possible involvement in antipsychotic drug effects and the genetic variants of SREBF1 and/or SREBF2 could affect schizophrenia susceptibility [44]–[45]. HapMap-based association study in a large German sample identified association between schizophrenia and five markers in SREBF1 and five markers in SREBF266. Additionally, scientists have demonstrated in glial cell lines that antipsychotic drugs induce the expression of genes involved in cholesterol and fatty acids biosynthesis through activation of the sterol regulatory element binding protein (SREBP) transcription factors, encoded by the sterol regulatory element binding transcription factor 1 (SREBF1) and sterol regulatory element binding transcription factor 2 (SREBF2) genes [45].

The results presented were limited by a modest sample size and required more samples to replicate. Quantitative reverse transcription-polymerase chain reaction were required to exam the expression levels of 48 genes, which were found differentially expressed in our pilot study, in a larger sample of SSD and MDD. Additional studies were required to further explore the roles of these 48 genes in pathophysiology of SSD and MDD.

In conclusion, our study demonstrated that SSD and MDD exhibited a unique expressed genome signature with peripheral blood leukocyte, and blood cell–derived RNA may have significant value for performing diagnostic functions and identifying disease biomarkers in SSD and MDD.

## Materials and Methods

The study was conducted at the Division of Mood Disorders, Shanghai Mental Health Center, Shanghai Jiao Tong University School of Medicine between Jan 2007 and Dec 2009. Outpatients were recruited from the clinic and ward of Shanghai Mental Health Center. All procedures were reviewed and approved by Institutional Review Boards of Shanghai Mental Health Center. Written informed consent was obtained from each subject before any study-related procedures were performed.

### Subjects

Inclusion criteria for SSD group were: two or more depressive symptoms for at least 2 weeks with social dysfunction but without depressed mood or anhedonia, and having a total score of 17-item Hamilton Rating Scale for Depression (HRSD-17) from 8 to 16. Patients were included into MDD group who met DSM-IV criteria for MDD and had the total score of HRSD-17 ≥17. Patients were excluded if they had substance dependence, severe medical illness, organic brain disease, pregnancy. Healthy control subjects have a score 7 or lower on the HRSD-17, and did not have any major Axis I disorders (including substance dependence, psychotic disorders, mood disorders and anxiety disorders), family history of mental disorder or severe physical diseases (hypertension, diabetes, cancer).

For the gene expression microarray analysis, this study enrolled eight drug-free Chinese Han patients with their first episode of subsyndromal symptomatic depression, eight previously untreated patients presenting with their first episode of major depression disorder, and eight healthy controls. All groups were matched with sex and age (shown in Table 5).

**Table 5:** Demographic data for patients and healthy controls.

Group	Age (years)	Gender	Course of disease(months)
SSD			
1	25	M	3.0
2	27	M	5.0
3	27	M	2.5
4	36	M	3.0
5	29	F	3.25
6	30	F	1.75
7	35	F	3.5
8	41	F	2.0
MDD			
1	24	M	3.3
2	26	M	5.3
3	27	M	2.5
4	38	M	3.0
5	28	F	3.5
6	31	F	1.5
7	35	F	3.0
8	41	F	2.25
HC			
1	24	M	-
2	29	M	-
3	27	M	-
4	37	M	-
5	28	F	-
6	30	F	-
7	35	F	-
8	41	F	-

MDD: Major depression disorder; SSD: Subsyndromal symptomatic depression; HC: Healthy controls.

All subjects were screened by the Structured Clinical Interview for DSM-IV (SCID) and assessed through HRSD-17 score by two experienced psychiatrists (inner coherence, Kappa = 0.87).

### Peripheral blood lymphocytes collection and RNA processing

Total 20 ml venous peripheral blood from fasting patients and healthy controls were collected during 7am to 9am. Peripheral blood lymphocytes were separated by Ficoll gradient centrifugation using Ficoll-PlaqueTM Plus (GE, Sweden) [46].Total RNA was extracted from lymphocytes using Trizol reagent (Invitrogen) according to the manufacturer's protocol. RNA quality was determined by Nanodrop ND-1000 (Nanodrop Technologies, Wilmington, DE) and degradation of mRNA was assessed by denaturing agarose gel electrophoresis and evaluated the sharpness of 28 S and 18 S rRNA bands.

### Microarray data pre-processing

24 samples were profiled on affymetrix U133 Plus2.0 GeneChip oligonucleotide arrays (Affymetrix, Santa Clara, CA), which is comprised of more than 22,000 probe sets and can analyze the expression level of 18,400 transcripts and variants (approximately 11,000 genes). The preparation of cRNA hybridization, signal scanning, data acquisition, and preliminary analysis were performed at the National Engineering Center for Biochip at Shanghai according to the standard protocols recommended by Affymetrix (Affymetrix, Santa Clara, CA, USA). Raw data generated from affymetrix Human U133Plus2.0 were processed and normalized by RMA method with Gene Spring Software 11.0 (Agilent technologies, Santa Clara, CA, US), then the values were log2 transformed. Differential gene analysis was preliminarily performed using Welch t test and then P value adjustment under multiple hypothesis testing was implemented with multtest package in Bioconductor under the adjustment method of Bonferroni. We used Welch t test and boost strap resampling approach (B = 100,000) to compute t statistics and p values. The threshold for differential expressed genes (DEGs) was chosen as 0.01.

All data is MIAME compliant and that the raw data has been deposited in a MIAME compliant database (E.g. ArrayExpress, GEO), as detailed on the MGED Society website http://www.mged.org/Workgroups/MIAME/miame.html. The accession numbers is GSE32280.

### Disease model and classification

To select the smallest size of biomarkers with robust predictive power and fewer potential false positives, more stringent thresholds were used to identify genes with even greater reliability. Firstly, the thresholds for differentially expressed SSD and MDD signatures compared to control and differentially expressed signatures between SSD and MDD were set as 1.0E-4, 5.0E-4 and 1.0E-4 respectively. Alternatively, P values in combination with fold-change values were used to identify potential biomarker genes to limit the likelihood of false positive results. Secondly, these signatures from 3 pair-wise comparisons were ranked according to their adjusted P values and the top N signatures were merged directly (to obtain a small size of biomarkers comparatively and a better classification performance, the top 10, 15, 18, 20, 25 and 30 signatures from each group were merged respectively). Then, we applied SVM (Support vector machines) on each of candidate expression profiles to search better combination of biomarkers with robust prediction performances (accuracy, sensitivity or specificity). Finally, leave-one-off method was used to validate the biomarkers. Leave-one-off validation involves using a single observation from the original sample as the validation data, and the remaining observations as the training data. This was repeated such that each observation in the sample was used once as the validation data.

### Gene Ontology Analysis

Standard methods for testing over-representation of a GO category assume that, under the null hypothesis, each gene has equal probability of being detected as DEG (differential expressed gene) [47]. Under this assumption, the number of genes associated with a category that overlap with the set of DEG follows a hypergeometric distribution. Hence the GO test can be conducted using Fisher's exact test, which uses the hypergeometric distribution, or Pearson's chi-square test, which is a computationally convenient approximation.

### Network and Pathway Analysis

Pathway was analysis using human pathways from KEGG, biocarta, and metabolism pathway databases [48]. Scoring the prioritation of network/pathways according to the relevance to input data. In cases of SSD and MDD experiments result, we analysis how different pathways and networks modules can be prioritized based on their statistical significance with respect to such experimental datasets. Significance is evaluated based on the size of the intersection between differential expressed gene signatures and set of genes/proteins corresponding to a network module/pathway curated in pathway database. This problem can be cast as selection without replacement and the probability to randomly obtain intersection of certain size between differential expressed gene signatures and a network/pathway follows hypergeometric distribution When considering a set of DEG signatures (I), invariable number r of DE signatures among the N nodes of the pathway/network module. The probability of a subset of size n to include r DE genes provided that n and R are unrelated (null-hypothesis) follows the hypergeometric distribution.

### Multiclass SVM implementation

In order to classify SSD and MDD from healthy control simultaneously, support vector machines (SVMs) was utilized for training and testing on candidate signature expression profiles from signature selection step. SVMs which represents an extension to nonlinear models of the generalized portrait algorithm developed by Vladimir Vapnik is a group of supervised learning methods that can be applied to classification or regression [49]. The SVM takes a set of input data, and predicts, for each given input, which of two possible classes the input is a member of, which makes the SVM a non-probabilistic binary linear classifier. Since an SVM is a classifier, then given a set of training examples, each marked as belonging to one of two categories, an SVM training algorithm builds a model that predicts whether a new example falls into one category or the other.

The original SSD, MDD and control problem may be stated in a finite dimensional space, but it often happens that in that space the sets to be discriminated are not linearly separable. For this reason it was proposed that the original finite dimensional space be mapped into a much higher dimensional space presumably making the separation easier in that space. In order to clearly classify SSD and MDD from controls, multiclass SVM were also used in aims to assign labels to instances by using support vector machines. The multiclass approach for conducting this is to reduce the single multiclass problem into multiple binary classification problems. Each of the problems yields a binary classifier, which is assumed to produce an output function that gives relatively large values. In end, polynomial kernel was applied with the best predictive performances for combinatorial gene signatures from the three groups.

### De novo cis-Regulatory element analysis

Cis-regulatory motifs are essential elements for gene transcription [50]. We also interrogated the over-representative motifs on promoter sequences collected from UCSC (www.genome.ucsc.edu/). Two thousand bps sequences around TSS for SSD and MDD signatures and biomarkers for classifying three groups (MDD, SSD and controls, together) were all considered for in promoter-based de novo motif analysis.
